# The efficacy of pregabalin for the management of postoperative pain in primary total knee and hip arthroplasty: a meta-analysis

**DOI:** 10.1186/s13018-017-0540-0

**Published:** 2017-03-24

**Authors:** Fei Li, Jianxiong Ma, Mingjie Kuang, Xuan Jiang, Ying Wang, Bin Lu, Xingwen Zhao, Lei Sun, Xinlong Ma

**Affiliations:** 1 Biomechanics Labs of Orthopaedics Institute, Tianjin Hospital, Tianjin, 300050 People’s Republic of China; 2 Tianjin Medical University, Tianjin, 300070 People’s Republic of China

**Keywords:** Pregabalin, Arthroplasty, Meta-analysis, Pain

## Abstract

**Objective:**

A systematic review of randomized controlled trials (RCTs) was conducted to evaluate the efficacy of pregabalin for the management of postoperative pain in patients undergoing primary total knee arthroplasty (TKA) and primary total hip arthroplasty (THA).

**Method:**

The PubMed, Embase, Cochrane Central Register of Controlled Trials, and Google Scholar databases were searched for related articles using search strategy. RevMan 5.3 software was selected to conduct the meta-analysis.

**Results:**

Seven RCTs were included in our meta-analysis. There were significant differences in visual analogue scale (VAS) at 24 and 48 h with rest, knee flexion degree, mean morphine consumption, and postoperative side effects (nausea, vomiting, pruritus, and dizziness) when comparing the pregabalin group to the placebo group after TKA and THA. However, the differences in VAS at 72 h with rest and at 24 h on movement were not significant between the two groups.

**Conclusions:**

Pregabalin was found to improve pain control at 24 and 48 h with rest, reduce morphine consumption, improve the knee flexion degree, decrease the incident rate of nausea, vomiting, and pruritus, and increase the incident rate of dizziness after TKA and THA but could not improve the pain control at 72 h with rest. In summary, the use of pregabalin may be a valuable asset in pain management within the first 48 h after TKA and THA. However, future studies regarding doses and pregabalin medication are required.

## Background

Total knee arthroplasty (TKA) and total hip arthroplasty (THA) have become common treatments for patients with severe knee and hip functional impairment. These surgeries can significantly relieve pain, restore joint function, and improve quality of life [1, 2]. The number of patients undergoing TKA and THA has increased steadily each year, and this trend will continue in the foreseeable future due to the aging of the population [3]. Postoperative pain after total joint arthroplasty has remained a serious problem, which prolongs length of hospital stay and functional recovery. Therefore, appropriate pain management protocol is necessary to relieve postoperative pain and achieve early functional recovery.

Opioids have generally been used for pain control after TKA or THA. However, common side effects, such as sedation, dizziness, nausea, vomiting, constipation, physical dependence, tolerance, and respiratory depression, are serious problems with these medications [4, 5]. Pregabalin is a structural analog of γ-aminobutyric acid (GABA) that acts on the α2δ subunit of voltage-dependent calcium channels, which can reduce the release of neurotransmitters [6–8]. Although it was approved for the treatment of partial seizures, pregabalin has been administered for other indications, such as fibromyalgia, diabetic neuropathy, and even acute postsurgical pain [9].

In recent years, some randomized controlled trials (RCTs) have been carried out to evaluate the effects of pregabalin. However, different conclusions have been reached, and the efficacy of pregabalin for pain management among TKA and THA patients has remained unclear. Jain et al. [10] reported that perioperative administration of pregabalin reduced opioid consumption, improved postoperative analgesia, and yielded higher patient satisfaction levels in primary TKA. However, Singla et al. [11] showed that there was no significant difference between pregabalin and placebo with respect to the worst pain (24/48 h postsurgery) after TKA. Similar conclusions have been reached by YaDeau [12]. Therefore, the role of pregabalin for pain control and functional recovery after TKA and THA has not been investigated in systematic review and meta-analysis. This meta-analysis was conducted to determine whether pregabalin used systematically can decrease postoperative pain and improve joint function when compared with placebo.

## Materials and method

### Search strategy

**Fig. 1:**
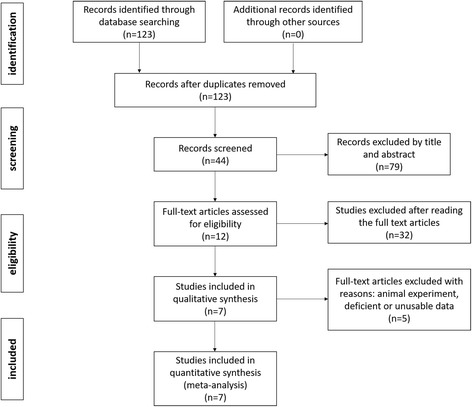
Search results and selection procedure

The PubMed (1980–July 2016), Embase (1980–July 2015), Cochrane Central Register of Controlled Trials, and Google Scholar databases were searched for related articles using the search strategy outlined in Appendix 1. According to the Cochrane Collaboration guidelines, we also conducted other database searches. The search strategy is presented in Fig. 1.

### Study selection

Included studies were considered eligible if they met the following criteria:

Study design: Interventional studies

Population: Patients were scheduled for primary TKA and THA

Intervention: Pregabalin

Comparator: Placebo or nothing

Outcome:

Primary outcome: Visual analogue scale (VAS) at 24, 48, and 72 h with rest and at 24 h on movement and morphine consumption

Secondary outcome: Knee flexion degree and treatment side effects (nausea, vomits, pruritus and dizziness)

### Quality assessment

**Fig. 2:**
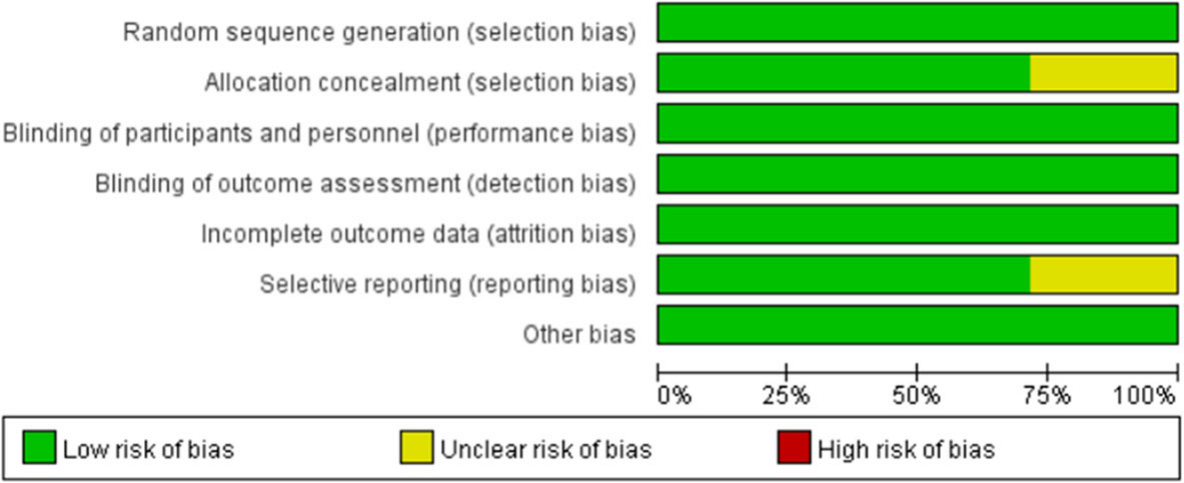
Risk of bias graph

**Fig. 3:**
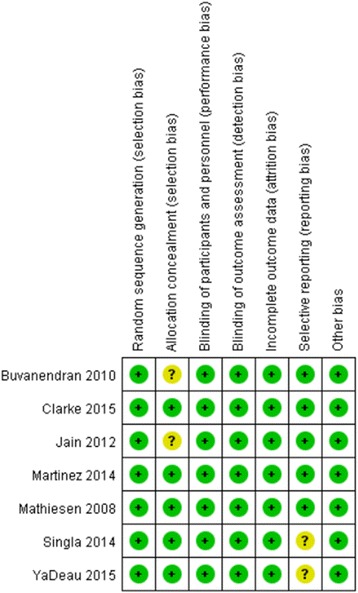
Risk of bias summary

Published RCTs comparing pregabalin with a control (placebo or nothing) in patients who underwent primary TKA or THA are included in this meta-analysis. The eligibility assessment was conducted by two reviewers (F.L. and JX.M.) independently in an unblended standardized manner. Disagreements were resolved by consensus. According to the Cochrane Handbook for Systematic Reviews of Interventions [13], the methodological quality and risk basis of the included studies were evaluated as follows: (1) randomization method, (2) allocation concealment, (3) blind method of participant and outcome assessment, and (4) complete outcome data. The risk of bias can be seen in Figs. 2 and 3.

### Data extraction

Data were extracted from the enrolled studies by two authors independently. The extracted data included publication data, title, first author’s name, patient demographics, sample size, morphine consumption, pain scores, knee flexion degree, postoperative complications, and side effects (nausea, vomiting, pruritus, and dizziness).

### Data analysis and statistical methods

The data were analyzed by Review Manager Software for Windows (RevMan Version 5.3, Copenhagen; The Nordic Cochrane Center, The Cochrane Collaboration, 2014). The means and standard deviations were applied to assess continuous variable outcomes with a 95% confidence interval (CI), and relative risks and 95% CIs were applied to assess dichotomous outcomes. Statistical heterogeneity was tested using the *I* ^2^ value and chi-squared test. A *P* value <0.05 was considered statistically significant, and the random effect model was used for analysis. If *P* values were <0.05 or *I* ^2^ > 50%, indicating significant heterogeneity, the random effect model was applied.

## Result

### Search result

A total of 123 relevant articles were identified in the databases. Seven RCTs were included by reading the abstracts and entire article in detail. The overall methodological quality of the included studies was relatively high. All of the RCTs applied randomized, placebo-controlled, and double-blind strategies, which reflected the high quality of the included literature. Baseline data were provided in all included studies without providing the intention to treat analysis. Finally, 823 patients were included in our meta-analysis. The patient characteristics are presented in Table 1. The sample sizes for each study ranged from 40 to 216.

**Table 1:** The general characteristic of the included studies

Studies	Cases (Pre/P)	Mean age (Pre/P)	Male patient (Pre/P)	Pregabalin dosage (mg/d)	Reference type	Postoperative analgesics
Buvanendran et al. [14]	106/110	64/63	29/36	300	RCT	PCEA + celecoxib 200 mg bid for 3 days while in the hospital
Jain et al. [10]	20/20	60/57	9/5	150	RCT	PCEA (containing bupivacaine 0.0625% and morphine 0.05 mg/ml)
Singla et al. [11]	96/98	64/63	35/44	300	RCT	PCA (morphine) + oral analgesia 5 mg hydrocodone bitartrate/500 mg acetaminophen tablets every 4–6 h
YaDeau et al. [12]	30/30	68/66	7/16	300	RCT	PCEA (morphine) + meloxicam 5 mg and oxycodone paracetamol 325 mg
Mathiesen et al. [15]	40/38	67/66	14/18	300	RCT	PCA (morphine) + acetaminophen 1 g tid
Clarke et al. [16]	83/79	60/60	41/41	150	RCT	PCA (morphine) for 24 h + celecoxib 200 mg bid, oxycontin 5 mg tid
Martinez et al. [17]	35/38	64/64	20/25	150	RCT	PCA for 48 h, then paracetamol, non-steroidal anti-inflammatory drugs and oral opioid

*Pre/P* pregabalin/placebo, *QAS* quality assessment score, *RCT* randomized controlled trial, *PCT* prospective control trials

Randomization was stated in all RCTs through a computer-assisted program, and all of the RCTs used a double-blind methodology. Four studies [10–12, 14] were for TKA, while another three studies [15–17] were for THA, and there were 510 patients for TKA and 313 patients for THA. All of the articles had been published since 2008, and four of the articles had been published in the last 3 years. In experimental groups, each patient received pregabalin for corresponding dosage 1–2 h before surgery, and patients received pregabalin with dosages ranging from 150 to 300 mg orally in the postoperative days. One TKA study used a dosage of 150 mg pregabalin every day after surgery, and three studies used a 300 mg dosage. YaDeau [12] conducted a multidose trial in which the dosage of pregabalin was 0, 100, 200, and 300 mg for each group every day. In this meta-analysis, we use the data of the 300 mg as the experimental group and that of the 0 mg as the control group. Singla [11] conducted a multicenter, randomized, double-blind, placebo-controlled trial; one part of the study was for TKA, two dosages (150, 300 mg/d) were included, and we used the 300 mg dosage for our meta-analysis. For THA studies, two studies used 150 mg/d pregabalin for the experimental group, and one study used 300 mg/d. Postoperative analgesia included patient-controlled analgesia/patient-controlled epidural analgesia (PCA/PCEA), celecoxib, acetaminophen, non-steroidal anti-inflammatory drugs, and morphine. Postoperative analgesia details can be seen in Table 1.

### Meta-analysis results

#### Morphine consumption

**Fig. 4:**
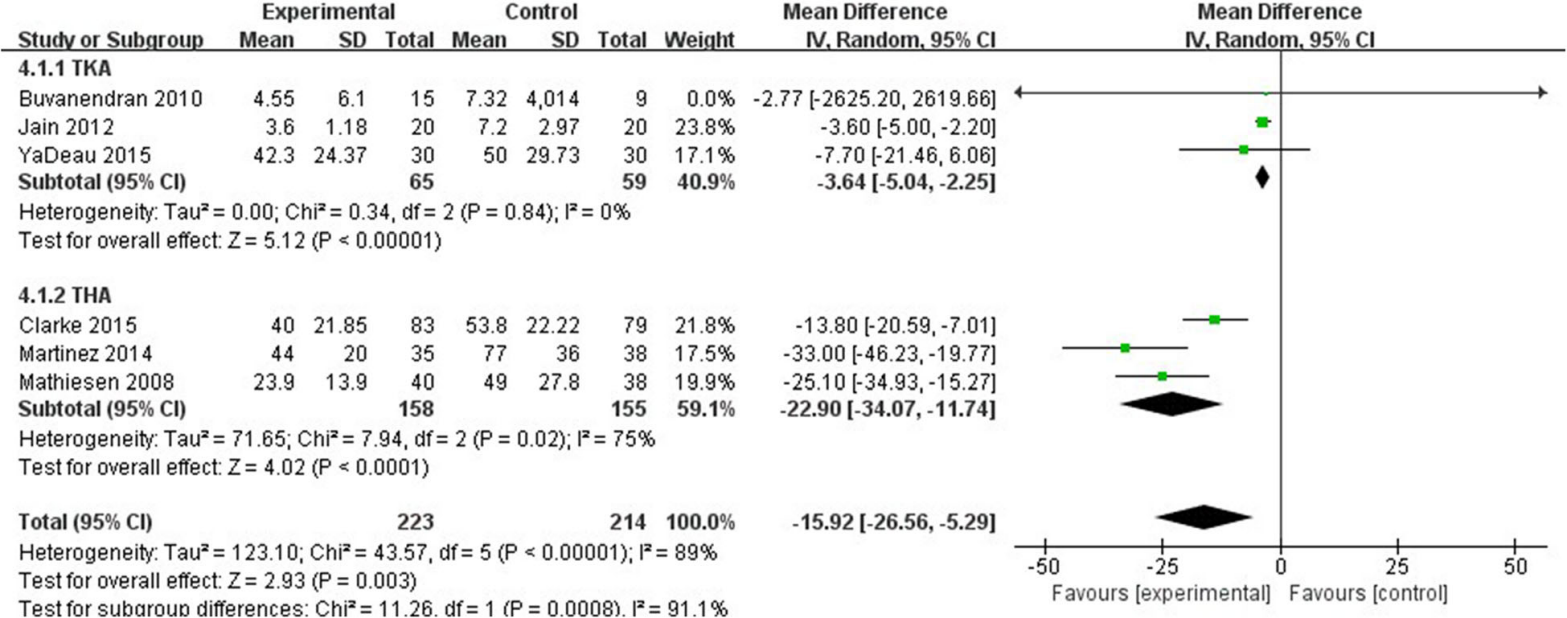
The effect of pregabalin illustrated by a forest plot diagram on mean morphine consumption

Based on six studies providing available data, we found that there was significant heterogeneity (*χ* ^2^ = 43.57, df = 5, *P* < 0.00001, *I* ^2^ = 89%). As depicted in Fig. 3, the pooled results produced a better outcome between the two groups according to a random effects model (MD = −15.92, 95% CI, [−26.56−5.29], *P* = 0.003, Fig. 4). A subgroup analysis was performed for the morphine consumption (Table 2).

**Table 2:** Subgroup analysis for morphine consumption

Subgroup or outcomes	Studies	Effect estimate			
		*χ* ^2^	MD and 95% CI	*I* ^*2*^(%)	*P*
TKA	3	0.34	−3.64(–5.04,−2.25)	0	0.84
THA	3	7.94	−22.90(−34.07,−11.74)	75	0.02

### VAS score at rest

#### VAS at 24 h

**Fig. 5:**
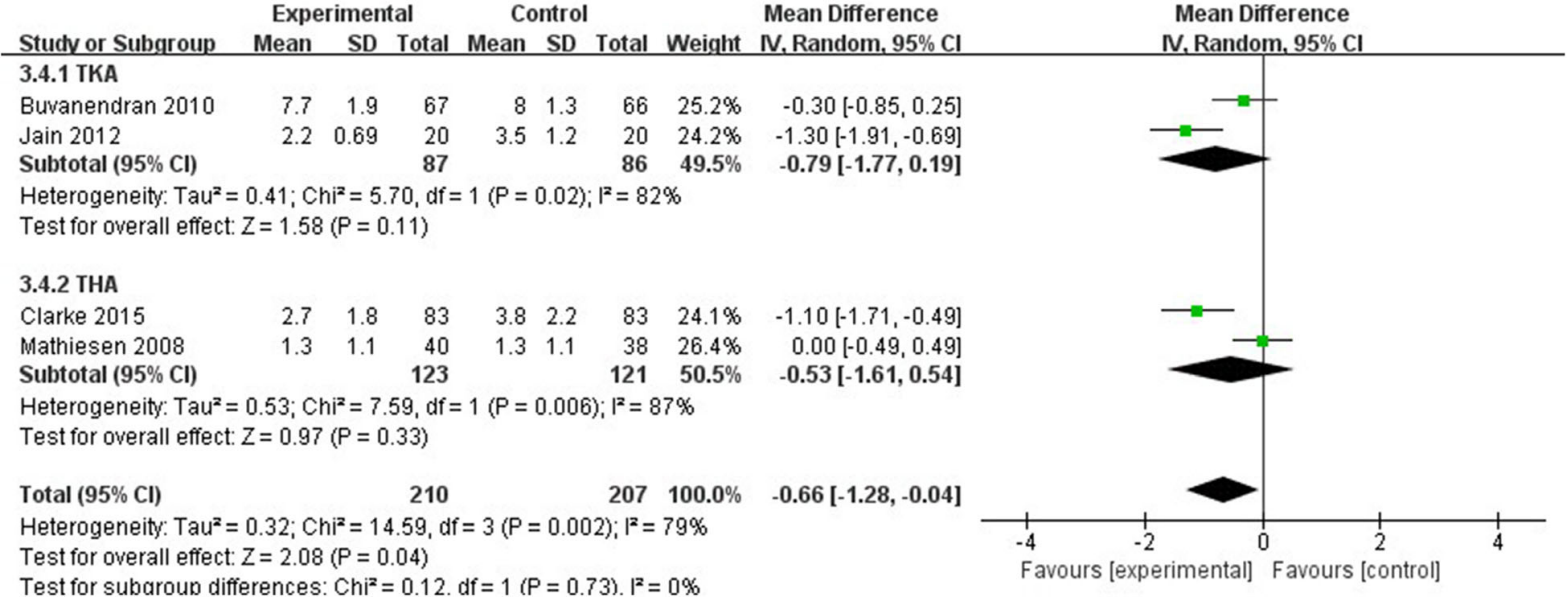
The effect of pregabalin illustrated by a forest plot diagram on 24 h VAS at rest

A total of four component studies with 417 patients provided a VAS score at 24 h after surgery with rest. Our meta-analysis revealed that pregabalin produced a better outcome compared to the control group with rest at 24 h in terms of VAS score (MD = −0.66, 95% CI [−1.28–0.04], *P* = 0.04, Fig. 5). We used a random effect model because statistical heterogeneity was high (χ^2^ = 14.59, df = 3, *P* = 0.002, *I* ^*2*^ = 79%).

#### VAS at 48 h

**Fig. 6:**
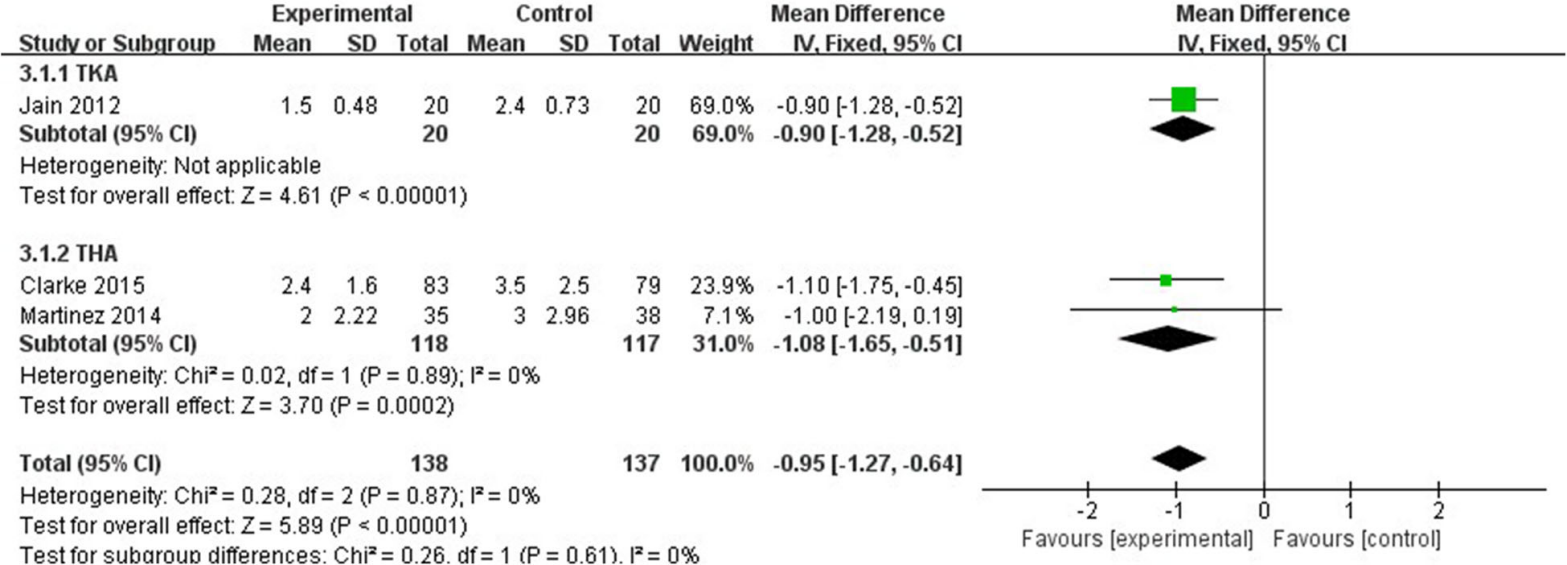
The effect of pregabalin illustrated by a forest plot diagram on 48 h VAS at rest

Only three studies with 275 patients reported the VAS score at 48 h postoperatively. Our meta-analysis found a highly significant difference between the two groups (MD = −0.95, 95% CI, [−1.27–0.64], *P* < 0.00001, Fig. 6). A fixed-effect model was preferred because the statistical heterogeneity was low (χ^2^ = 0.28, df = 2, *P* = 0.87, *I* ^2^ = 0%).

#### VAS at 72 h

**Fig. 7:**
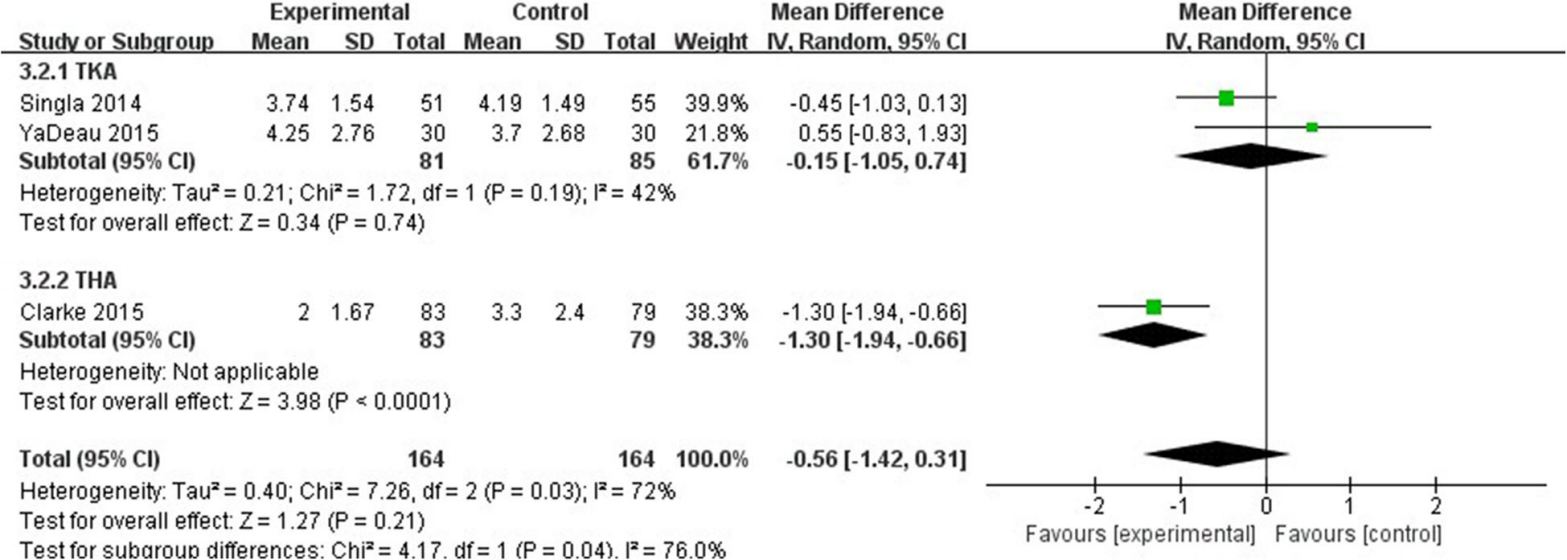
The effect of pregabalin illustrated by a forest plot diagram on 72 h VAS at rest

Three studies stated the VAS score at 72 h postoperatively with rest, and our meta-analysis revealed that there was no significant difference between the two groups (MD = −0.56, 95% CI, [−1.42–0.31], *P* = 0.21, Fig.7). We used a random effect model because of the significant statistical heterogeneity (*χ* ^2^ = 7.26, df = 2, *P* = 0.03, *I* ^2^ = 72%).

### VAS on movement

**Fig. 8:**
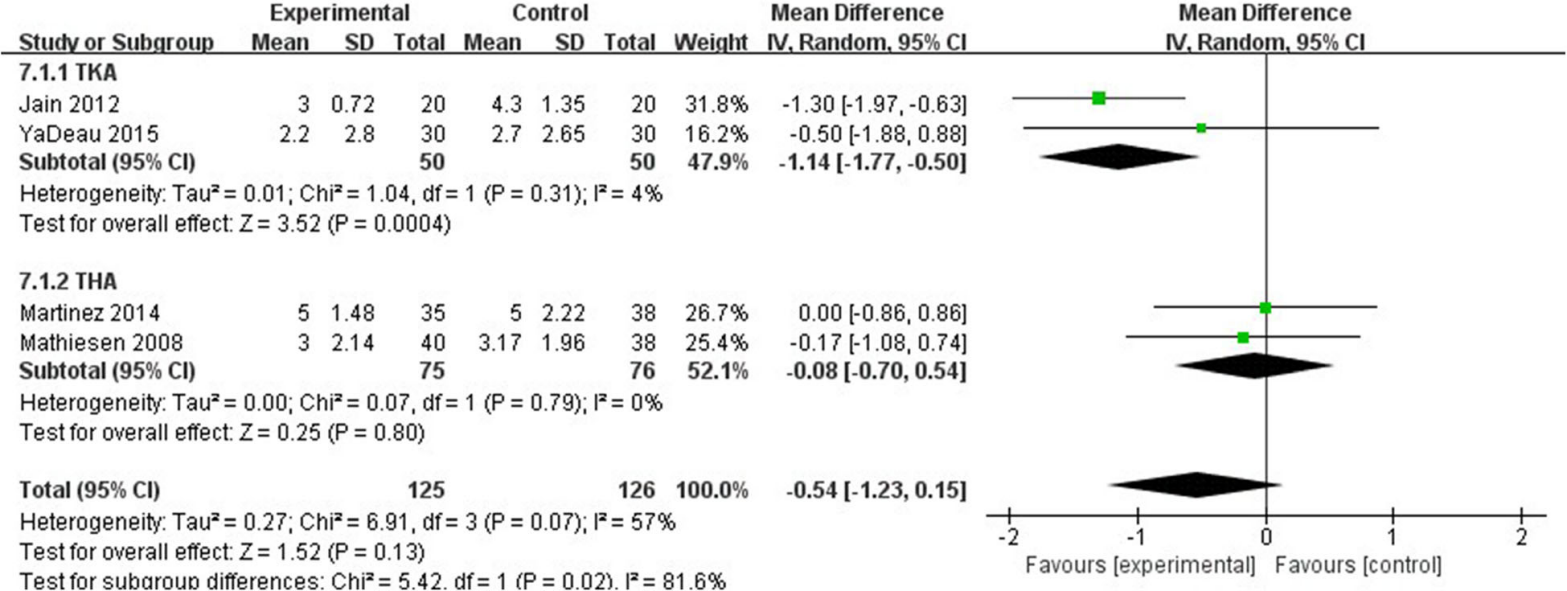
The effect of pregabalin illustrated by a forest plot diagram with 24 h VAS on movement

Four studies in our meta-analysis reported the VAS score on movement at 24 h, and the available data demonstrated that there was no significant difference between the two groups (MD = −0.54, 95% CI, [−1.23–0.15], *P* = 0.13, Fig. 8). The pooled results showed significant heterogeneity (*χ* ^2^ = 6.91, df = 3, *P* = 0.07, *I* ^2^ = 57%), and therefore, a random effect model was used.

### Knee flexion degree

**Fig. 9:**
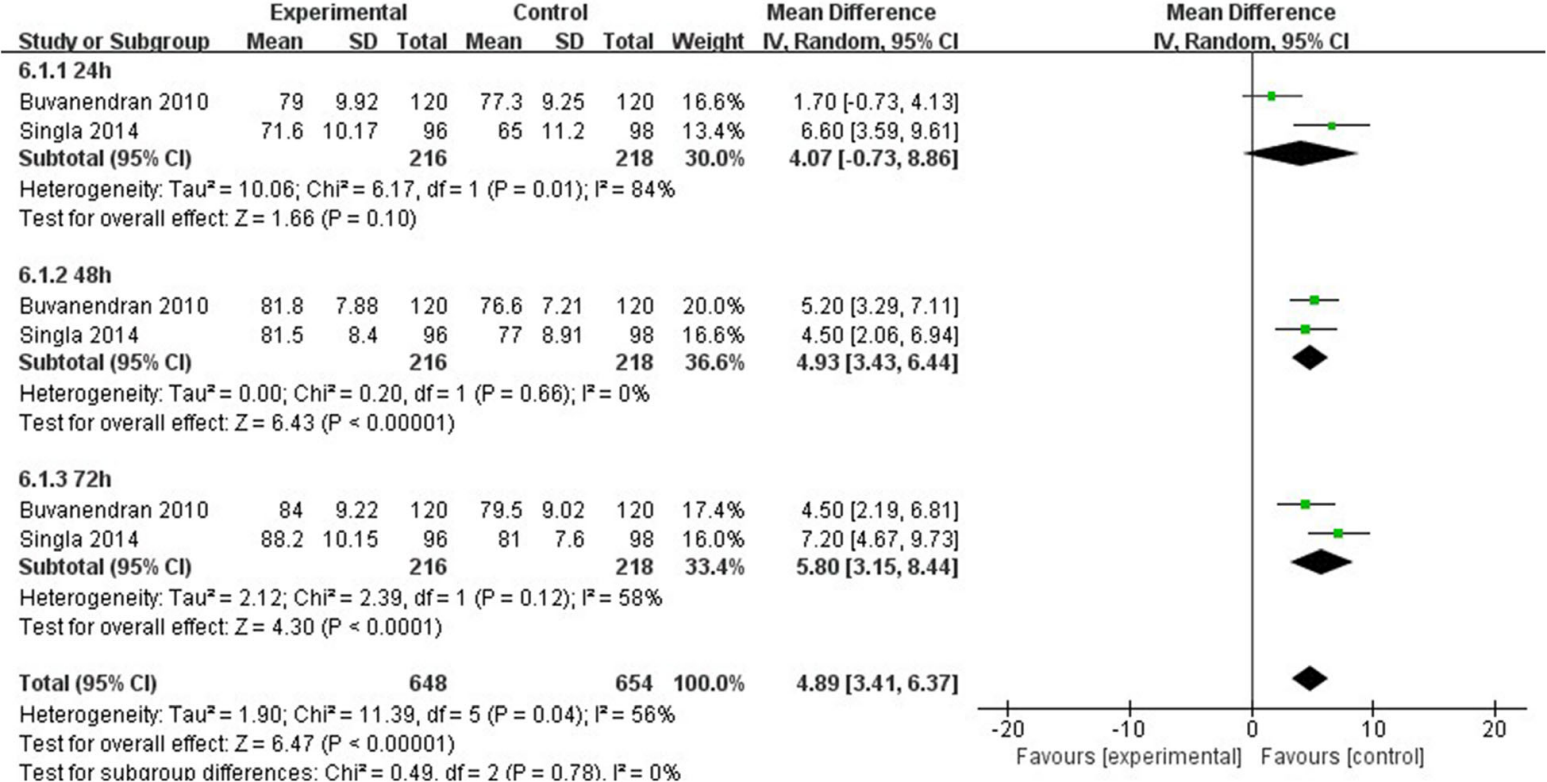
The effect of pregabalin illustrated by a forest plot diagram on knee flexion degree

Only two studies [11, 14] provided the data of knee flexion degree measured at 24, 48, and 72 h, respectively. The results demonstrated that there were significant differences between the two groups in TKA patients (MD = 4.89, 95% CI, [3.41, 6.37], *P* < 0.00001, Fig. 9). There was significant heterogeneity between the two groups based on the pooled data (*χ* ^2^ = 11.39, df = 5, *P* = 0.04, *I* ^2^ = 56%), so we used a random effect model.

### Side effect

**Fig. 10:**
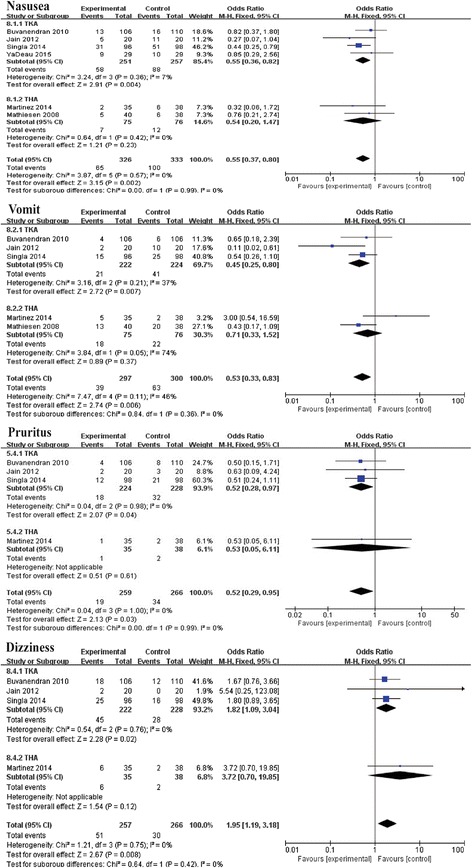
The effect of pregabalin illustrated by a forest plot diagram on side effects (nausea, vomiting, pruritus, and dizziness)

We summarized four side effects of pregabalin after TKA and THA (Fig. 10). The results are presented in Table 3.

**Table 3:** Results of side effect

Outcome	Studies	Overall effect			Heterogeneity	
		Effect estimate	95% CI	*P* value	*I* ^*2*^ (%)	*P* value
Nausea	6	0.55	0.37, 0.80	0.002	0	0.57
Vomits	5	0.53	0.33, 0.83	0.006	46	0.11
Pruritus	4	0.52	0.29, 0.95	0.03	0	1.0
Dizziness	4	1.95	1.19, 3.18	0.008	0	0.75

## Discussion

This meta-analysis was performed to systematically review the literature and form a comprehensive understanding of the efficacy of pregabalin for the management of postoperative pain after TKA or THA. Our results indicated that the pregabalin played an important role in the VAS with rest at 24 and 48 h, knee flexion degree, morphine consumption, and adverse events. No statistically significant differences were found in the VAS score with rest at 72 h and VAS on movement at 24 h between the pregabalin group and the control group.

Mean morphine consumption was one of the primary outcomes in our study. Six studies documented mean morphine consumption, and significant difference was found between the pregabalin group and the control group in our meta-analysis (*P* < 0.003). We converted opioid use to morphine equivalents in all studies because different opioid drugs and units of measurement were used to record opioid consumption. It was very important to reduce opioid consumption after surgery because some patients would be easily addicted to opioid medications used after TKA or THA [18]. Our pooled data showed that pregabalin could decrease postoperative opioid consumption. This result agrees with those of other studies examining the administration of pregabalin [10, 14, 19]. Another meta-analysis showed that pregabalin can reduce 24-h morphine consumption in gynecologic, laparoscopic cholecystectomy, orthopedic, spine, and miscellaneous procedures [20]. These outcomes indicate that ideal analgesic effects can be achieved with less morphine consumption when pregabalin is used simultaneously.

The VAS pain score was a useful indicator for the assessment of pain with high value. A 10-cm-long moveable ruler was used to test the pain level for patients after TKA or THA (0–>10, 0 was no pain and 10 was unbearable pain). In our study, 24, 48, and 72 h at rest and 24 h of movement postoperatively were selected as the point-in-time for comparison. This meta-analysis showed significant difference between 24 and 48 h VAS scores at rest, which indicates that pregabalin can effectively relieve postoperative pain at rest. Jokela et al. [21] found that premedication with 75 or 150 mg pregabalin significantly reduced the VAS score for postoperative pain at 24 h after laparoscopic gynecologic surgery (*P* < 0.05) when compared with diazepam 5 mg. Some other studies [22, 23] have reported that pregabalin administration could reduce the VAS score at 24 h compared with placebo postoperatively. Gianesello [24] showed that perioperative administration of pregabalin at a dose of 300 mg every day resulted in a significant reduction in VAS scores at 48 h postoperatively for patients undergoing major spinal surgery. These outcomes demonstrate that administration of pregabalin could reduce VAS score at 24 and 48 h with rest after surgery. The VAS at 72 h with rest postoperatively was not significant in our pooled data. The decreased benefits of pregabalin on pain during movement could be explained by the short mean elimination half-life of pregabalin [25]. No significant difference was found in VAS on movement at 24 h, and the result was similar to another meta-analysis of pregabalin for tonsillectomy [26]. One explanation for this could be that the movement of body intensified the pain after surgery. Additionally, the sample size was small, with only four included studies. Therefore, we could not determine the effect of pregabalin for TKA and THA at 24 h of movement postoperatively. Although no significant reduction in VAS score at 72 h with rest and at 24 h on movement postoperatively, the score of the pregabalin group was lower than that of the control group in most studies. In summary, preoperative administration of pregabalin could improve acute pain control after TKA and THA.

Knee flexion degree was a valuable indicator to evaluate the motion of knee joints and functional recovery [27, 28]. We established a subgroup to investigate the reasons of heterogeneity and the data of different time periods. Our pooled data showed that the pregabalin groups can enhance the knee range of motion effectively (*P* < 0.00001). Buvanendran [14] demonstrated that pregabalin shortened the time to achieve normal knee range of motion (ROM). He thought this beneficial effect on knee functions at the time of discharge facilitated nearly full functionality. Pregabalin was shown to improve pain control, and less pain can lead to more extensive activities, which may be the reason for the improvement of knee function. Singla [11] showed that passive knee range of motion for the operated knee was greater for the 300 mg/d pregabalin group compared with placebo at 24, 72, 96, 120 h, and at week 4 post-TKA, and active ROM (upon flexion) was significantly greater in the 300 mg/d pregabalin group compared with that of the placebo only group at week 4. Thus, according to this result, we concluded that perioperative pregabalin consumption had beneficial effects on improving knee function.

Postoperative nausea and vomiting were usually caused by different anesthetic modes that were mostly related to the gastrointestinal system [29]. Our pooled data showed that postoperative oral pregabalin could decrease the incidence of nausea and vomiting. The same results were reported in another meta-analysis [30], which indicated that preoperative pregabalin is associated with a significant reduction of postoperative nausea and vomiting. Based on these outcomes, postoperative administration of pregabalin has been shown to reduce nausea and vomiting. Pruritus is another side effect during the administration of pregabalin. As shown in Fig. 10, pregabalin can significantly decrease the incident rate of postoperative pruritus. Şavk [31] suggested that pregabalin at a daily dose of 150 mg was effective in reducing chronic pruritus. Dizziness was the most common adverse effect profile of pregabalin. The pooled data showed that the pregabalin would increase the incident rate of dizziness. Griffin [32] reported that dizziness, fatigue, and somnolence were among the most common adverse effects of pregabalin. In summary, we found that pregabalin can effectively reduce the incidence of nausea, vomiting, and pruritus, but it can increase the incidence of dizziness.

Our meta-analysis has the following potential limitations: (1) only seven RCTs were selected in our meta-analysis; if more studies were included, statistical efficacy would increase. (2) The follow-up period of patients was too short in some of the trials. Most patients were followed up only in the short term. This may have resulted in underreporting of some useful information. (3) There were not sufficient data, such as neuropathic pain, sleep disturbance, time to discharge, and physical composite score above. (4) Risk of bias cannot be avoided in this meta-analysis because only English publications were included. (5) Almost all the included studies were published by anesthetists, and some important details usually considered by orthopedics, such as surgical approach, methods of fixation, technique of incision, and type of implant were not reported in those papers. It is believed that all of these factors have the ability to change the degree of postoperative pain, and that they need to be taken into account in future studies.

Although this study has several limitations, it is the first systematic review to evaluate the efficacy of pregabalin with placebo in primary TKA and THA. Available articles were screened strictly; hence, the articles adopted for the final review were of high quality. However, more high-quality literature should be included to elevate statistical efficacy and increase sample size.

## Conclusions

Our meta-analysis indicated that pregabalin could improve pain control at 24 and 48 h with rest, reduce morphine consumption, and improve the knee flexion degree as well as decreasing the incident rate of nausea, vomiting, and pruritus and increasing the incident rate of dizziness after TKA and THA but could not improve the pain control at 72 h with rest. In summary, the use of pregabalin may be a valuable asset in pain management within first 48 h after TKA and THA. However, future studies regarding doses and pregabalin medication are required.

## Appendix 1

#1 Total Knee Replacement

#2 Total Knee Arthroplasty

#3 “Arthroplasty, Replacement, Knee” [Mesh]

#4 Search (#1 OR #2) OR #3

#5 Total Hip Replacement

#6 Total Hip Arthroplasty

#7 “Arthroplasty, Replacement, Hip” [Mesh]

#8 Search (#5 OR #6) OR #7

#9 Search (#4 OR #8)

#10“Pregabalin” [Mesh]

#11 random*

#12 “Randomized Controlled Trial” [Publication Type]

#13 “Randomized Controlled Trials as Topic” [Mesh]

#14 Search (#11 OR #12 OR #13)

#15 Search (#9 AND #10 AND # 14)

## Abbreviations

CI: Confidence interval
GABA: γ-Aminobutyric acid
RCT: Randomized controlled trial
ROM: Range of motion
THA: Total hip arthroplasty
TKA: Total knee arthroplasty
VAS: Visual analogue scale
